# Failure of early lymphocyte recovery identifies sepsis patients with initial lymphopenia at highest risk for late mortality

**DOI:** 10.1371/journal.pone.0353698

**Published:** 2026-07-17

**Authors:** Binli Ma, Gan Lin, Jinhu Li, Lian Xie, Jiale Yang, Huasheng Tong

**Affiliations:** 1 Guangdong Pharmaceutical University, Guangzhou, China; 2 Department of Critical Care Medicine, General Hospital of the Southern Theatre Command of the People’s Liberation Army of China, Guangzhou, China; 3 The First School of Clinical Medicine, Southern Medical University, Guangzhou, China; 4 Department of Critical Care Medicine, Chinese PLA General Hospital, Beijing, China; Pescara General Hospital, ITALY

## Abstract

**Background:**

Sepsis-induced lymphopenia is associated with adverse outcomes, yet static assessments at a single time point fail to capture the clinical significance of its dynamic evolution, and the effects of the trajectories of lymphopenia on prognosis are to be elucidated in sepsis.

**Methods:**

This retrospective cohort study extracted data from the MIMIC-IV (v3.1) database. Adult septic patients with initial lymphopenia (<1.0 × 10⁹/L) were included. Group-Based Trajectory Modeling (GBTM) identified lymphocyte count trajectories. Kaplan-Meier analysis with log-rank test was used to visualize survival differences. Multivariable Cox and logistic regression assessed associations between trajectories and 7-day, 28-day, ICU, and in-hospital mortality. Subgroup analyses validated result consistency.

**Results:**

A total of 1,640 patients were included. GBTM identified 4 distinct lymphocyte count trajectories. Kaplan-Meier analysis revealed that patients in Trajectory 4 (Persistent Low Level, N = 544, 33.2%), characterized by persistent lymphopenia, had the lowest cumulative 7-day and 28-day survival rates. After multivariable adjustment, Trajectory 4 was independently associated with an increased risk of 28-day mortality [adjusted HR = 1.76, 95% CI: 1.266–2.446, P = 0.001], ICU mortality (adjusted OR = 1.831, 95% CI: 1.196–2.832, P = 0.006), and in-hospital mortality (adjusted OR = 1.881, 95% CI: 1.298–2.767, P = 0.001) compared to Trajectory 1 (Sustained Recovery), and these associations remained robust across all predefined subgroups. In contrast, no significant association was observed between Trajectory 4 and 7-day mortality after full adjustment for confounders.

**Conclusion:**

Lymphocyte count Trajectory 4 (Persistent Low Level) in initially lymphopenic sepsis patients is independently associated with late (28-day) but not early (7-day) mortality, as well as worse ICU and hospital outcomes. Failure of lymphopenia to recover by days 3–4 may signal a transition to the highest-risk state, suggesting a potential window for immunomodulatory strategies.

## Introduction

Sepsis is a common life-threatening condition in the intensive care unit (ICU) [1]. According to global data in 2017, the annual incidence of sepsis exceeded 48 million cases, with the related mortality accounting for nearly 20% of all global deaths [2]. The pathophysiology of sepsis is characterized by a complex interplay of systemic inflammation and immune dysregulation [3].Lymphopenia and lymphocyte dysfunction are often considered the key hallmarks of immunosuppression in sepsis [4].A cut-off of lymphocyte count below 1.0 × 10^9^/L has been widely established as a key biomarker and threshold for identifying patients at high risk of immunosuppression in sepsis [5–8].Practically, profiling lymphocyte count dynamics is crucial for deciphering disease progression, predicting prognosis, and guiding therapeutic development in sepsis.

Previous studies have established that dynamic monitoring of lymphocyte counts holds significant prognostic value in critical illnesses including sepsis, as it captures the evolution of the immune status more precisely. For example, while the lymphocyte count at admission to ICU is not significantly associated with 28-day mortality in shock patients, persistent lymphopenia over 3 days shows significant prognostic value [9]. Similarly, Drewry et al. have found that persistent lymphopenia on the fourth day following the diagnosis of sepsis predicts early and late mortality and may serve as a biomarker for immunosuppression in sepsis [10].Furthermore, longitudinal studies have identified distinct lymphocyte count trajectory phenotypes linked to clinical outcomes in sepsis [11,12]. Li et al. have reported that patients in the lymphocyte count trajectory(high-declining) within 3 days had the highest disease severity and mortality rate of 25.9% in sepsis [13]. Our previous work indicated that the lymphocyte count trajectory (rapid-slow increasing) is a strong predictor of 7- and 28-day mortality in sepsis [14].

However, the previous studies primarily analyzed lymphocyte count trajectories in the whole septic individuals. Furthermore, although initial lymphopenia (<1.0 × 10⁹/L), a key baseline indicator for assessing immune status [15], explicitly defines a high-risk subgroup of poor prognosis in septic patients [16], no study has specifically observed their dynamic nature and association with prognosis in the initial lymphopenic population in sepsis. This study aimed to characterize the 7-day lymphocyte count dynamics, uncover heterogeneous trajectory phenotypes, and assess their predictive values for clinical outcomes in sepsis patients with initial lymphopenia using the previously validated Group-Based Trajectory Modeling (GBTM) [14,17], which possibly provided a scientific foundation for precision risk stratification and potential immunomodulatory interventions in this high-risk population.

## Materials and methods

### Data source

Data for this retrospective cohort study were extracted from the Medical Information Mart for Intensive Care IV (MIMIC-IV, version 3.1) database. This public, de-identified database, jointly maintained by the Beth Israel Deaconess Medical Center (BIDMC) and the Massachusetts Institute of Technology (MIT), contains comprehensive medical records of over 70,000 ICU patients admitted to BIDMC between 2008 and 2022. It includes detailed demographics, vital signs, laboratory results, comorbidities, treatments, and outcomes, enabling robust analyses of sepsis. The Institutional Review Boards of both MIT and BIDMC approved the use of the database for research and waived the requirement for informed consent due to the de-identified nature of the data. One of the authors (Gan Lin) completed the required CITI Program training and was granted access to the database under Record ID: 64590357. Data access for research purposes was initiated on July 27, 2024 (the date when database access authorization was granted). Given the de-identified nature of the MIMIC-IV database (with all individual patient identifiers removed), no author had access to information that could identify participants during or after data collection. This study adheres to the STROBE statement [12] and the principles of the Declaration of Helsinki.

### Inclusion and exclusion criteria

**Fig 1:**
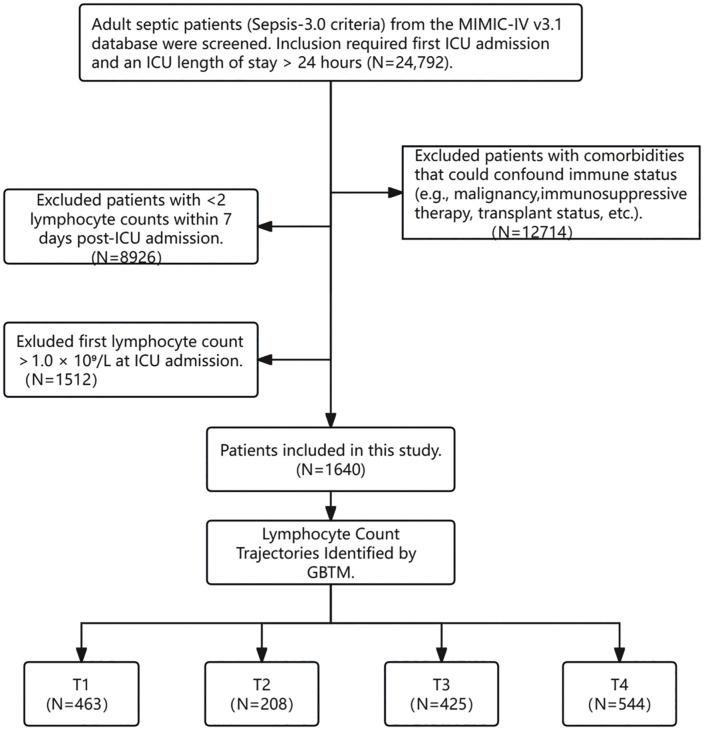
The flow chart for patient screening.

Sepsis was diagnosed using Sepsis-3.0 criteria: presence of documented or clinically suspected infection, accompanied by an acute infection-related increase in Sequential Organ Failure Assessment (SOFA) score ≥ 2 points [18].The inclusion criteria of patients were as follows:(1) patients who were diagnosed with sepsis, (2) patients aged ≥ 18 years old,(3) patients were first admitted to the ICU, and (4) ICU length of stay was > 24 hours (to ensure sufficient time for collecting baseline and follow-up data).The exclusion criteria were as follows:(1)<2 lymphocyte count measurements within 7 days after ICU admission (insufficient data for dynamic trajectory analysis),(2)initial lymphocyte count ≥1.0 × 10⁹/L within 24 hours of ICU admission (the minimum value was adopted if multiple measurements were available), and(3) presence of immune/hematopoietic interfering factors including cancer, metastatic solid tumor, anti-cancer therapy, acquired immunodeficiency syndrome (AIDS), long-term steroid use, transplant status, rheumatic disease, or hematologic disease. A total of 1,640 patients who met the diagnostic criteria for sepsis were enrolled. (Fig 1).

### Variable extraction

The key variables were extracted and categorized from MIMIC-IV v3.1. Data included: (1) Demographics: age and sex;(2) Comorbidities: myocardial infarction, congestive heart failure, chronic pulmonary disease, liver disease, diabetes, arrhythmia, and acute kidney injury (AKI); (3) Vital Signs: heart rate, respiratory rate, systolic blood pressure (SBP), diastolic blood pressure (DBP), mean arterial pressure (MAP), and temperature;(4) Laboratory Values: white blood cell count (WBC), monocytes, neutrophils, platelets, hematocrit, hemoglobin, albumin, blood urea nitrogen (BUN), calcium, creatinine, glucose, sodium, potassium, international normalized ratio (INR), prothrombin time (PT), alanine aminotransferase (ALT), alkaline phosphatase (ALP), aspartate aminotransferase (AST), lactate, and pH; (5) Severity Scores: Charlson Comorbidity Index (CCI), Acute Physiology Score III (APS III), Logistic Organ Dysfunction System (LODS), Oxford Acute Severity of Illness Score (OASIS), Simplified Acute Physiology Score II (SAPS II), and Sequential Organ Failure Assessment (SOFA); (6) Interventions: mechanical ventilation, glucocorticoids (administered over 3 or 7 days), continuous renal replacement therapy (CRRT), vasopressors, and sedatives. For all variables except lymphocyte count, the first value within 24 hours after ICU admission was used (the mean was calculated if multiple values were available). The minimum lymphocyte count for each day within the first 7 days after admission was extracted.A complete list of all abbreviations is provided in S1 Table.

### Outcomes

The primary outcomes were 7-day and 28-day all-cause mortality, and secondary outcomes included ICU mortality and in-hospital mortality.

### Statistical analysis

GBTM was employed to identify distinct subgroups of patients based on their longitudinal lymphocyte count patterns within the first 7 days after ICU admission. GBTM, a specialized finite mixture model that classifies individuals into unobserved (latent) trajectories, is more robust and objective than pre-defined subjective criteria for capturing the heterogeneity of variables.

The modeling process was performed using the lcmm package in R. To determine the optimal number of trajectories, we fitted a series of models and compared their fit using the Bayesian Information Criterion (BIC) and Akaike Information Criterion (AIC) [19]. Model selection also considered clinical interpretability and the requirement that each trajectory group must comprise at least 5% of the study population. The adequacy of the final model was evaluated using the average posterior probability (AvePP) of group membership. An AvePP > 0.7 for all groups was considered indicative of good internal reliability. Patients were subsequently assigned to the trajectory group for which they had the highest posterior probability.As shown in Supplementary S2 table, the four-trajectory model achieved the optimal balance between statistical goodness-of-fit and clinical interpretability, and was therefore selected as the final model.

The baseline characteristics were stratified by the identified GBTM trajectories.Variables with a missing rate of more than 20% were excluded, and detailed information on the missing data rates of all variables is presented in S3 Table. For variables with less than 20% missing data, multiple imputation was performed using the mice package in R software (version 4.3.1).

The Kolmogorov-Smirnov test was used to assess the normality of continuous variables. Normally distributed variables were expressed as mean ± standard deviation and compared across the four trajectory groups using one-way ANOVA. Non-normally distributed variables were expressed as median (interquartile range, IQR) and compared using the Kruskal-Wallis test. Categorical variables were expressed as frequency (percentage) and compared using the Chi-square test or Fisher’s exact test, as appropriate.

The association between the identified trajectory groups and the primary outcome was visualized using Kaplan-Meier survival curves and compared with the log-rank test.

For the primary outcomes, multivariable Cox proportional hazards regression analysis was employed to calculate hazard ratio (HR) with 95% confidence interval (CI). For secondary outcomes, multivariable logistic regression was used to calculate odds ratio (OR) with 95% CI. Covariates for Model 2 and Model 3 were selected based on clinical relevance, established literature, and our prior work. A hierarchical adjustment strategy was used to assess the stability of the effect estimates and to avoid over-adjustment.To mitigate the risk of over-adjusting for potential confounders, we constructed three sequential models:

Model 1 (Unadjusted)

Model 2: Adjusted for demographics and clinical characteristics (sex, age, weight, mean arterial pressure), illness severity scores (SOFA, APS III, LODS, OASIS, SAPS II, Charlson Comorbidity Index), and comorbidities (myocardial infarction, congestive heart failure, chronic pulmonary disease, liver disease, diabetes, arrhythmia, and AKI stage).

Model 3 (Fully Adjusted): Adjusted for all variables in Model 2, plus laboratory parameters (white blood cell count, neutrophils, platelets, hemoglobin, albumin, blood urea nitrogen, creatinine, glucose, INR, lymphocyte count, lactate, pH) and treatments (mechanical ventilation, glucocorticoid use within 3 and 7 days).

The variance inflation factor (VIF) was calculated for all covariates in the fully adjusted models to assess multicollinearity. The detailed VIF values for all covariates are presented in S4 Table, and all VIF values were below 10, indicating no significant multicollinearity.

Subgroup analyses were conducted to validate the associations between lymphocyte trajectory groups and 28-day mortality. Stratification was performed by sex, age, comorbidities, and critical illness scores. Interaction terms between the trajectory groups and these stratification factors were tested, with a P-value for interaction < 0.1 considered indicative of a statistically significant interaction effect.

All statistical analyses were performed using R software (version 4.3.1) and DecisionLine version 1.0. A two-sided p-value ≤ 0.05 was considered statistically significant for all analyses, unless otherwise specified.

## Results

### Lymphocyte Trajectory Subgroups Identified by GBTM

**Fig 2:**
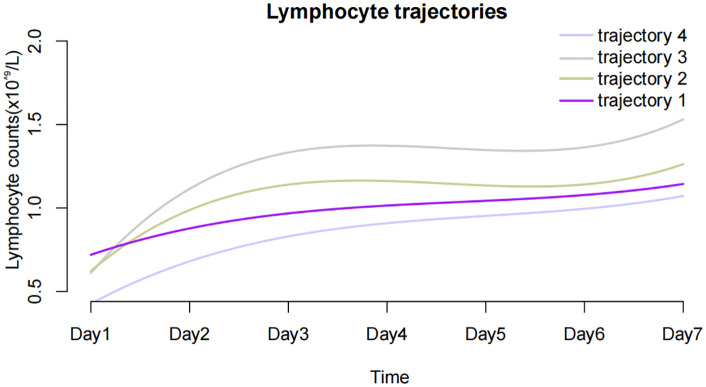
Lymphocyte count trajectories within 7 days after ICU admission.

This study aimed to elucidate dynamic lymphocyte patterns in septic patients with initial lymphopenia. We derived our cohort from the MIMIC-IV v3.1 database by applying stringent criteria, as depicted in Fig 1, excluding patients with conditions that could confound immune status (e.g., cancer, transplant status, or long-term glucocorticoid use). The final cohort comprised 1,640 patients. Using GBTM, we identified 4 distinct trajectories of lymphocyte count over the first 7 days following admission: Trajectory 1 (T1, Sustained Recovery), Trajectory 2 (T2, Rapid Recovery-Low Fluctuation), Trajectory 3 (T3, Rapid Recovery-High Fluctuation), and Trajectory 4 (T4, Persistent Low Level) (Fig 2). T1 (463, 28.2%) had the highest baseline lymphocyte count, featuring a stable, low-fluctuation upward trend and recovery to normal levels early (≥1.0 × 10^9^/L). T2 (208, 12.7%), starting from a moderately decreased baseline, showed a rapid increase with minimal fluctuation, reaching normal levels around day 3. T3 (425, 25.9%), starting from a moderately decreased baseline with a comparable initial slope to T2, was distinguished by significant day-to-day fluctuations. T4 (544, 33.1%), the most distinct subgroup, had the lowest lymphocyte count baseline, exhibited no meaningful recovery, and remained persistently lymphopenic.

In summary, while all patients shared an initial state of lymphopenia, they exhibited significant differences in lymphocyte recovery kinetics. Although T1, T2, and T3 varied in the stability of lymphocyte counts, all achieved effective recovery within 3–4-day window after admission. However,T4 was characterized by persistent lymphopenia, highlighting its unique clinical profile and underscoring its potential link to adverse outcomes.

### Baseline Characteristics and Clinical Outcomes of the Study Cohort

The baseline characteristics, interventions, comorbidities, and clinical outcomes of the entire cohort (N = 1,640) and the four trajectory subgroups are summarized in Table 1. For the overall cohort, the median age was 67.07 years, 58.72% were male, and the median baseline lymphocyte count was 0.59 × 10⁹/L,consistent with the criterion of initial lymphopenia. Regarding the primary outcomes, the overall 28-day and 7-day all-cause mortality rates were 19.39% (318/1,640) and 10.18% (167/1,640), respectively. For the secondary outcomes, the overall ICU and hospital mortality rates were 15.18% (249/1,640) and 20.61% (338/1,640), respectively.

**Table 1:** Baseline Characteristics and Clinical Outcomes of the Study Cohort.

Characteristics	T1 (N = 463)	T2 (N = 208)	T3(N = 425)	T4(N = 544)	p-value
**Baseline variables**					
**Age** ^*^ **(years)**	64.38(53.72-78.49)	60.51(48.14-70.52)	69.55(57.78-82.41)	70.45(59.66-81.74)	<0.001
**Sex**					<0.001
Female**	216.00(46.65%)	101.00(48.56%)	149.00(35.06%)	211.00(38.79%)	
Male**	247.00(53.35%)	107.00(51.44%)	276.00(64.94%)	333.00(61.21%)	
**Vital signs**					
Weight*(kg)	81.60(67.00-100.00)	84.30(66.85-100.69)	79.30(67.00-95.00)	78.30(65.60-93.00)	0.055
Heart rate***(bpm)	89.80 ± 16.63	93.51 ± 18.45	87.36 ± 17.07	89.03 ± 17.86	<0.001
Resp rate*,bpm	20.83(18.08-23.73)	21.16(18.50-25.32)	20.44(18.09-23.08)	20.69(17.63-23.68)	0.045
SBP*(mmHg)	112.24(103.97-124.42)	108.88(101.41-116.47)	110.74(103.13-124.04)	110.78(102.99-120.15)	0.007
DBP*(mmHg)	62.38(56.50-68.71)	60.53(54.38-67.34)	61.42(55.27-68.68)	60.07(54.49-66.97)	0.009
MBP*(mmHg)	75.90(70.50-83.50)	73.88(69.01-81.52)	74.73(69.61-82.28)	74.12(68.38-80.53)	0.005
Temperature*(℃)	36.94(36.63-37.33)	36.99(36.64-37.52)	36.89(36.60-37.30)	36.86(36.52-37.30)	0.006
**Laboratory parameters**					
WBC*(10^9/L)	12.43(8.90-17.90)	13.83(9.70-20.95)	12.20(8.63-16.00)	10.58(7.12-14.87)	<0.001
Lymphocyte*(10^9/L)	0.64(0.40-0.82)	0.58(0.39-0.80)	0.72(0.60-0.85)	0.41(0.28-0.57)	<0.001
Monocytes*(10^9/L)	0.52(0.29-0.80)	0.50(0.27-0.86)	0.53(0.35-0.79)	0.44(0.26-0.68)	<0.001
Neutrophils*(10^9/L)	10.55(7.14-15.64)	11.31(7.29-18.18)	10.58(7.02-14.53)	8.80(5.51-13.43)	<0.001
Platelets*(10^9/L)	197.50(150.75-269.00)	201.25(117.83-274.88)	196.33(145.00-261.50)	166.50(119.10-230.00)	<0.001
Hematocrit***(%)	34.68 ± 5.71	34.78 ± 6.46	34.23 ± 6.51	34.12 ± 6.24	0.359
Hemoglobin***(g/dL)	11.37 ± 1.94	11.43 ± 2.14	11.21 ± 2.18	11.12 ± 2.14	0.155
Albumin*(g/dL)	3.15(2.86-3.41)	3.10(2.60-3.40)	3.16(2.90-3.47)	3.10(2.85-3.40)	0.111
BUN*(mg/dL)	23.00(15.50-37.33)	27.00(14.63-46.42)	27.33(16.67-41.33)	28.00(18.50-47.93)	<0.001
Calcium***(mg/dL)	8.22 ± 0.85	7.99 ± 0.76	8.22 ± 0.74	8.08 ± 0.71	<0.001
Creatinine*(mg/dL)	1.10(0.80-1.70)	1.30(0.90-2.20)	1.20(0.83-1.96)	1.30(0.93-2.00)	0.002
Glucose*(mg/dL)	141.00(116.00-176.40)	138.79(111.45-190.50)	138.75(113.00-183.00)	138.10(110.00-175.00)	0.342
Sodium*(mmol/L)	138.25(135.60-141.00)	138.00(135.29-140.67)	138.67(135.33-141.60)	138.67(135.00-141.92)	0.503
Potassium*(mmol/L)	4.13(3.80-4.60)	4.05(3.78-4.50)	4.17(3.80-4.63)	4.15(3.80-4.63)	0.502
INR*	1.30(1.10-1.57)	1.35(1.20-1.65)	1.33(1.20-1.73)	1.35(1.17-1.80)	0.005
PT*(s)	14.35(12.60-17.20)	14.90(13.35-18.05)	14.70(13.05-18.70)	15.05(12.94-19.70)	0.003
ALT*(U/L)	44.00(21.00-98.00)	36.17(19.58-86.33)	35.50(19.00-92.00)	39.00(20.58-97.75)	0.141
ALP*(U/L)	94.50(67.67-135.50)	89.50(64.00-118.00)	93.00(70.00-133.25)	94.75(66.75-137.13)	0.223
AST*(U/L)	56.50(30.00-141.00)	52.00(29.75-126.00)	54.00(28.00-121.50)	59.00(32.00-153.83)	0.377
Lactate*(mg/dL)	2.04(1.50-2.79)	2.16(1.70-3.01)	2.00(1.33-2.65)	2.12(1.60-2.81)	0.003
PH*	7.36(7.31-7.40)	7.34(7.29-7.38)	7.36(7.32-7.40)	7.34(7.30-7.38)	<0.001
**Score system**					
SOFA*	3.00(2.00-4.00)	4.00(2.50-5.00)	3.00(2.00-4.00)	3.00(2.00-5.00)	<0.001
APSIII*	49.00(38.00-62.00)	57.00(44.50-74.00)	53.00(43.00-67.00)	55.00(43.00-69.00)	<0.001
LODS*	5.00(3.00-7.00)	6.00(4.00-9.00)	6.00(4.00-8.00)	6.00(4.00-9.00)	<0.001
OASIS***	35.23 ± 8.51	36.85 ± 9.41	36.33 ± 8.63	37.24 ± 8.39	0.003
SAPSII*	37.00(30.00-47.00)	40.00(32.00-50.00)	40.00(34.00-51.00)	42.00(34.00-52.00)	<0.001
CCI*	4.00(2.00-6.00)	3.00(2.00-6.00)	5.00(3.00-7.00)	5.00(3.00-7.00)	<0.001
**Interventions**					
Ventilation**	411.00(88.77%)	184.00(88.46%)	384.00(90.35%)	509.00(93.57%)	0.034
Glucocorticoids3day**	179.00(38.66%)	80.00(38.46%)	127.00(29.88%)	225.00(41.36%)	0.002
Glucocorticoids7day**	160.00(34.56%)	66.00(31.73%)	113.00(26.59%)	208.00(38.24%)	0.002
CRRT*	25.00(5.40%)	28.00(13.46%)	21.00(4.94%)	46.00(8.46%)	<0.001
VP*	237.00(51.19%)	127.00(61.06%)	199.00(46.82%)	310.00(56.99%)	0.001
SA*	262.00(56.59%)	134.00(64.42%)	243.00(57.18%)	330.00(60.66%)	0.183
**Comorbidities**					
Myocardial infarct*	78.00(16.85%)	27.00(12.98%)	86.00(20.24%)	107.00(19.67%)	0.096
Congestive heart failure*	151.00(32.61%)	66.00(31.73%)	179.00(42.12%)	218.00(40.07%)	0.004
Chronic pulmonary disease*	159.00(34.34%)	59.00(28.37%)	137.00(32.24%)	180.00(33.09%)	0.490
Liver disease*	402.00(86.83%)	171.00(82.21%)	390.00(91.76%)	481.00(88.42%)	0.005
Diabetes*	142.00(30.67%)	58.00(27.88%)	153.00(36.00%)	172.00(31.62%)	0.161
Arrhythmia*	377.00(81.43%)	169.00(81.25%)	338.00(79.53%)	443.00(81.43%)	0.869
AKI*	302.00(65.23%)	147.00(70.67%)	318.00(74.82%)	412.00(75.74%)	0.002
**Primary outcome**					
7-daymortality*	34.00(7.34%)	17.00(8.17%)	45.00(10.59%)	71.00(13.05%)	0.019
28-daymortality*	58.00(12.53%)	34.00(16.35%)	78.00(18.35%)	148.00(27.21%)	<0.001
**Secondary outcome**					
ICU mortality*	47.00(10.15%)	30.00(14.42%)	62.00(14.59%)	110.00(20.22%)	<0.001
Hospital mortality*	65.00(14.04%)	35.00(16.83%)	84.00(19.76%)	154.00(28.31%)	<0.001

*Median (interquartile range, IQR); * *n (%); * * *Mean ± standard deviation.

### Demographics and Disease Severity

Patients in the T4 subgroup were the oldest (median age 70.45 years) and had the highest proportion of individuals aged≥65 years (63.60%). In terms of disease severity, patients in the T4 subgroup showed the highest SAPSII (median 42) and CCI (median 5). Although APS III(median 55) in T4 was slightly lower than that in T2 subgroup (median 57), both T4 and T2 exhibited comparably high SOFA scores.

### Laboratory Parameters

T4 subgroup had the lowest baseline lymphocyte counts (median 0.41 × 10⁹/L) at ICU admission.

### Comorbidities and Interventions

T4 subgroup had a significantly higher prevalence of congestive heart failure (CHF) (218/544, 40.07%) and AKI (412/544, 75.74%). Regarding treatment interventions, T4 subgroup showed the highest rate of mechanical ventilation (509/544, 93.57%). Although the utilization rates of vasopressors (310/544, 56.99%) and CRRT (46/544, 8.46%) in the T4 subgroup were numerically lower than those in the T2 subgroup (vasopressors: 127/208, 61.06%,and CRRT: 28/208, 13.46%), they remained at elevated levels.

### Clinical Outcomes Across Trajectory Subgroups

Significant differences in mortality were observed among the trajectory subgroups (Table 1). A clear gradient in unadjusted mortality rates was evident from T1 (lowest) to T4 (highest). Specifically, the 28-day mortality rates were 12.53% (58/463) for T1, 16.35% (34/208) for T2, 18.35% (78/425) for T3, and 27.21% (148/544) for T4. This graded pattern was consistent across all measured outcomes, including 7-day, ICU, and hospital mortality.

### Association Between Lymphocyte Trajectories and Outcomes

**Fig 3:**
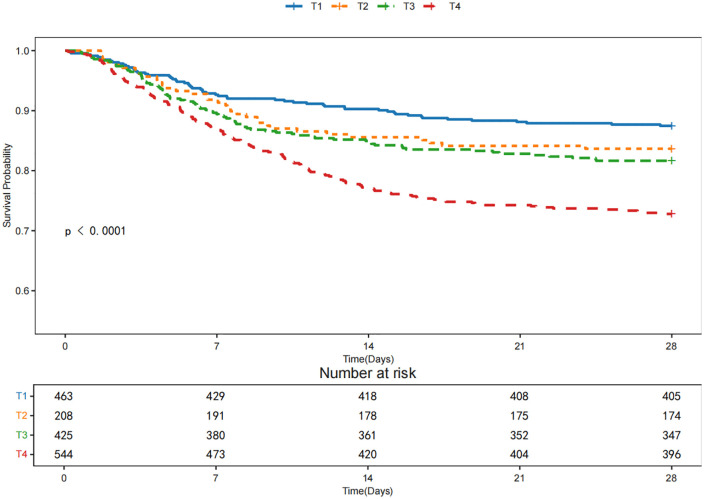
Kaplan-Meier curves extending to 28 days for the same cohort.

Kaplan-Meier analysis showed a significant association between lymphocyte count trajectories and mortality. During the 28-day follow-up period, significant differences were observed in the survival rates among 4 trajectory groups (log-rank test, P < 0.0001) (Fig 3). Pairwise comparisons indicated that the T4 subgroup had significantly lower 28-day survival than the T1 subgroup (P < 0.001), T2 subgroup (P = 0.003), and T3 subgroup (P = 0.002), demonstrating the poorest survival prognosis. The differences in 7-day survival rates among groups were statistically significant (log-rank test, P = 0.018), with T4 subgroup showing significantly lower survival than T1 subgroup (P = 0.003) (S1 Fig).

Survival curves were compared using the log-rank test. The p-value indicates the significance of the overall difference among the four groups across the entire follow-up period.

### Multivariable Regression Analysis

Multivariable Cox proportional hazards models were constructed to assess the independent impact of lymphocyte trajectories on mortality risk, with detailed results presented in Table 2. In Model 1(unadjusted), both the T3 (HR 1.519, 95% CI 1.081–2.133) and T4 (HR 2.348, 95% CI 1.733–3.181) subgroups showed a significantly increased 28-day mortality compared to the T1 subgroup (P < 0.001, Table 2). After sequential adjustment in Models 2 and 3, the T4 trajectory remained an independent risk factor for 28-day mortality. In the fully adjusted Model 3, the risk of death in the T4 subgroup (HR 1.76, 95% CI 1.266–2.446, P = 0.001) was 76% higher than that in the T1 subgroup (Table 2). The risks associated with the T2 and T3 subgroups became non-significant after adjustment.

**Table 2:** Cox regression analyzed lymphocyte trajectories and 7 – and 28-day mortality.

Outcome	Model 1		Model 2		Model 3	
	**HR (95%CI)**	**P-value**	**HR (95%CI)**	**P-value**	**HR (95% CI)**	**P-value**
**28-day mortality**						
**T1**	Ref (Reference)		Ref(Reference)		Ref(Reference)	
**T2**	1.336(0.875–2.040)	0.18	1.113(0.722–1.717)	0.627	1.004 (0.643–1.568)	0.986
**T3**	1.519 (1.081–2.133)	0.016	1.190 (0.841–1.684)	0.326	1.144 (0.800–1.638)	0.461
**T4**	2.348 (1.733–3.181)	<0.001	1.791 (1.317–2.437)	<0.001	1.760 (1.266–2.446)	0.001
**7-day mortality**						
**T1**	Ref (Reference)		Ref (Reference)		Ref (Reference)	
**T2**	1.120 (0.626–2.005)	0.703	0.777 (0.427–1.413)	0.408	0.686 (0.368–1.279)	0.236
**T3**	1.465 (0.938–2.286)	0.093	1.288 (0.816–2.033)	0.277	1.305 (0.812–2.097)	0.271
**T4**	1.836 (1.220–2.763)	0.004	1.474 (0.972–2.236)	0.068	1.481 (0.951–2.306)	0.082

Model 1 (Unadjusted); Model 2: Adjusted for demographics and clinical characteristics (sex, age, weight, mean arterial pressure), illness severity scores (SOFA, APS III, LODS, OASIS, SAPS II, Charlson Comorbidity Index), and comorbidities (myocardial infarction, congestive heart failure, chronic pulmonary disease, liver disease, diabetes, arrhythmia, and AKI stage); Model 3 (Fully Adjusted): Adjusted for all variables in Model 2, plus laboratory parameters (white blood cell count, neutrophils, platelets, hemoglobin, albumin, blood urea nitrogen, creatinine, glucose, INR, lymphocyte count, lactate, pH) and treatments (mechanical ventilation, glucocorticoid use within 3 and 7 days).

For 7-day mortality, the pattern of risk association changed markedly after adjustment. In the unadjusted Model 1, the T4 subgroup showed a significantly higher risk of death (HR 1.836, 95% CI 1.220–2.763, P = 0.004, Table 2). However, after sequential adjustment in Models 2 and 3, the strength of the association attenuated and was no longer statistically significant (HR 1.481, 95% CI 0.951–2.306, P = 0.082, Table 2).

For ICU and hospital mortality, multivariable logistic regression analyses showed a pattern highly consistent with the results about the independent impact of lymphocyte trajectories on 28-day mortality. In the fully adjusted Model 3, the risks of both ICU (OR 1.831,95%CI 1.196–2.832, P = 0.006) and hospital mortality (OR 1.893,95%CI 1.298–2.767, P = 0.001) in T4 subgroup were approximately 1.8 times higher than those in T1 subgroup (Table 3).

**Table 3:** Multivariable logistic regression analyzed lymphocyte trajectories in relation to ICU and hospital mortality.

Outcome	Model 1		Model 2		Model 3	
	**OR (95% CI)**	**P-value**	**OR (95% CI)**	**P-value**	**OR (95% CI)**	**P-value**
**ICU mortality**						
**T1**	Ref(Reference)		Ref(Reference)		Ref(Reference)	
**T2**	1.492(0.906–2.425)	0.11	1.125 (0.652–1.911)	0.667	1.048 (0.590–1.833)	0.872
**T3**	1.512(1.011–2.274)	0.045	1.336 (0.867–2.068)	0.191	1.375 (0.872–2.180)	0.172
**T4**	2.243(1.564–3.263)	<0.001	1.841 (1.247–2.750)	0.002	1.831 (1.196–2.832)	0.006
**Hospital mortality**						
**T1**	Ref (Reference)		Ref (Reference)		Ref (Reference)	
**T2**	1.239 (0.785–1.928)	0.349	1.003 (0.609–1.626)	0.992	0.939 (0.557–1.557)	0.810
**T3**	1.508 (1.059–2.156)	0.023	1.229 (0.840–1.801)	0.289	1.226 (0.822–1.830)	0.321
**T4**	2.418 (1.760–3.353)	<0.001	1.898 (1.348–2.693)	<0.001	1.881 (1.298–2.767)	0.001

Model 1 (Unadjusted): Model 2: Adjusted for demographics and clinical characteristics (sex, age, weight, mean arterial pressure), illness severity scores (SOFA, APS III, LODS, OASIS, SAPS II, Charlson Comorbidity Index), and comorbidities (myocardial infarction, congestive heart failure, chronic pulmonary disease, liver disease, diabetes, arrhythmia, and AKI stage).Model 3 (Fully Adjusted): Adjusted for all variables in Model 2, plus laboratory parameters (white blood cell count, neutrophils, platelets, hemoglobin, albumin, blood urea nitrogen, creatinine, glucose, INR, lymphocyte count, lactate, pH) and treatments (mechanical ventilation, glucocorticoid use within 3 and 7 days).

### Subgroup analysis

**Fig 4:**
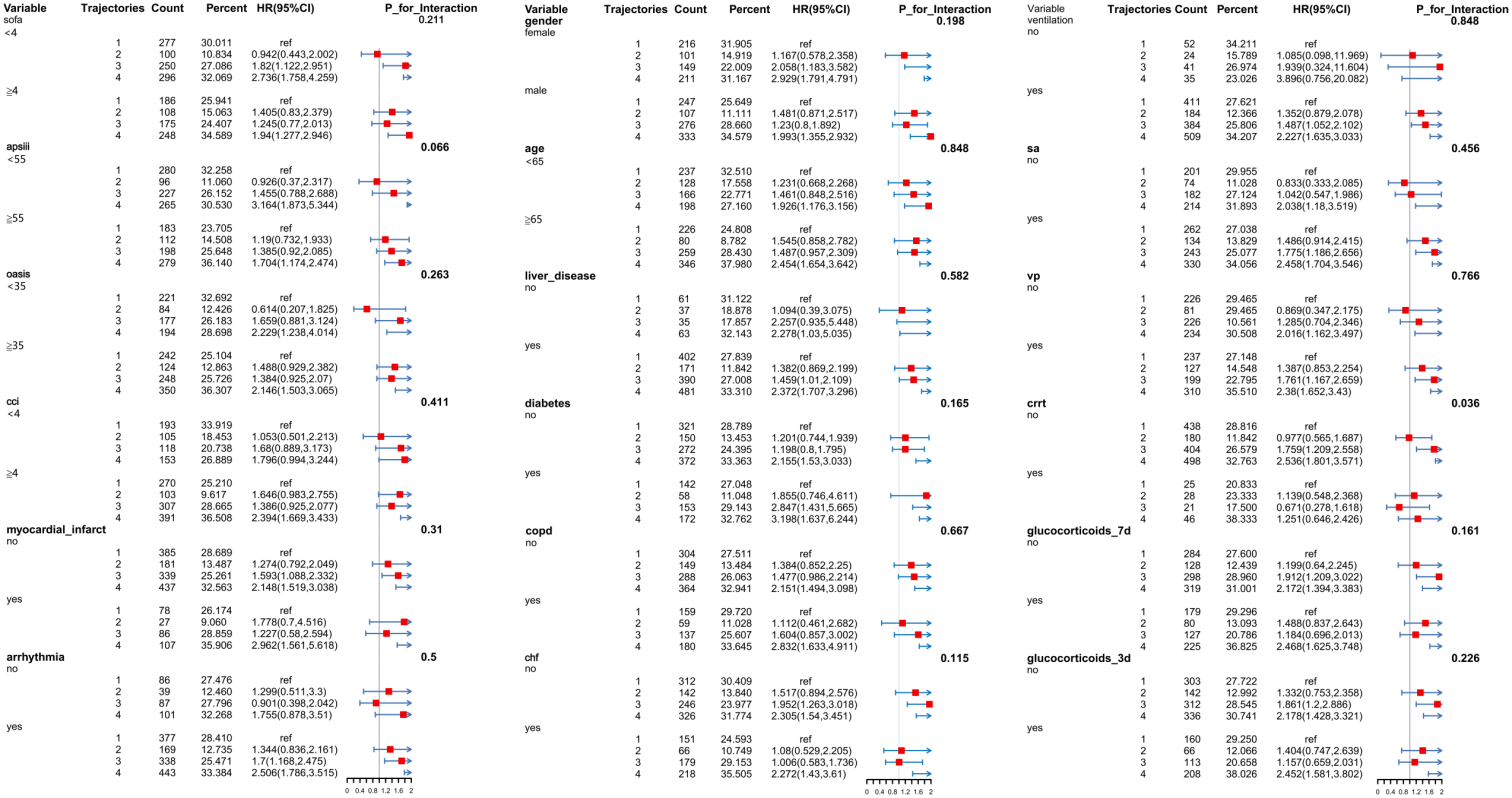
Subgroup analysis of the association between lymphocyte trajectories and 28-day mortality.

We performed subgroup analyses for the primary outcome of 28-day mortality (Fig 4) and the secondary outcomes of ICU and in-hospital mortality (S2-3 Fig). For all three outcomes, no significant interaction effects were observed between Trajectory 4 (persistent lymphopenia) and any of the stratification variables, including age, sex, comorbidities, disease severity scores, or interventions (all P for interaction > 0.05). This indicates that these baseline and clinical factors did not modify the association between the lymphocyte trajectory and patient prognosis.

## Discussion

This study is the first to delineate the dynamic landscape of early lymphocyte count trajectories and their association with clinical outcomes specifically in a high-risk sepsis subpopulation with initial lymphopenia (<1.0 × 10⁹/L) using GBTM. Our principal findings are threefold: (1) We successfully identified 4 distinct lymphocyte trajectories,T1 (Sustained Recovery), T2 (Rapid Recovery-Low Fluctuation), T3 (Rapid Recovery-High Fluctuation), and T4 (Persistent Low Level), demonstrating significant heterogeneity. (2) T4 emerged as an independent risk factor for 28-day mortality (HR = 1.77), ICU mortality (OR=1.84), and in-hospital mortality (OR=1.89) compared to the T1 trajectory, (3) T4 trajectory demonstrated a significant predictive value for 28-day but not for 7-day mortality after comprehensive adjustment for confounders. These results indicate that beneath a uniform “high-risk” label of initial lymphopenia, fundamental differences exist in the evolutionary path of the immune status, and the “Persistent Low Level” trajectory accurately identifies the immunophenotype with the poorest prognosis.

This discovery shifts the paradigm of our understanding of septic immunopathology from a static “initial state” to a dynamic “recovery capacity.” Although initial lymphopenia is an established risk marker, our research demonstrates that early recovery kinetics carry more profound prognostic information than a single baseline value. Patients capable of early lymphocyte count recovery (T1, T2, and T3), irrespective of the speed or stability of this recovery, exhibited superior long-term survival chances. Conversely, the “persistent immunoparalysis” phenotype, represented by the T4 subgroup, is an independent driver of long-term mortality risk. This explains why, even after excluding patients with overt confounding factors like malignancy or transplant, baseline characteristics alone are insufficient to fully discriminate outcomes.

Lymphocyte count is an important and readily available clinical indicator that reflects immune status and has been integrated into various risk scoring tools. For instance, the developed LIP scoring system, combining lymphocyte count, International Normalized Ratio (INR), and Procalcitonin (PCT), demonstrates high sensitivity (92.8%) and specificity (94.1%) for sepsis screening, and is superior to any single indicator [20].Similarly, combining the Neutrophil-to-Lymphocyte Ratio (NLR) with PCT, IL-6, or clinical scores like APACHE II significantly enhances the assessment of critical sepsis and prognostic prediction accuracy [21]. It has also become a core enrollment criterion for targeting immunomodulatory therapies in sepsis [22]. These studies confirm the crucial role of this static threshold for initial screening of high-risk populations. However, studies based on single-point values cannot capture the dynamic evolution of the immune system during the sepsis course [23]. This study employed the GBTM model to quantify the dynamic trajectories of lymphocyte counts over 7 days, revealing the critical impact of “persistent lymphopenia vs. recovery” differences in immune response on prognosis in sepsis.

In our previous study, dynamic analysis of lymphocyte counts using GBTM model showed that lymphocyte trajectories (e.g., Trajectory 3, “low level with slow increase”) could predict both 7-day and 28-day mortality and possibly reflected different immune phenotypes (pro-inflammatory injury, immunosuppression, immune stability), independently impacting short-term and late-term outcomes [14]. In the general patient population, a natural distribution exists between “normal” and “reduced” lymphocyte counts, and trajectories can effectively distinguish differences in immune phenotypes. In the present study, we focused on the high-risk patients with initial lymphopenia, and clearly identified T4 as the distinct phenotype with the worst prognosis.

Differences in baseline lymphocyte counts possibly represent the varying severity of the initial insult to the immune system. In contrast, the subsequent trajectory reflects the recovery capacity of the immune system better. Patients in T1, T2, and T3 groups achieved lymphocyte recovery early, indicating they were in a state of transient immunosuppression and possibly had good recovery potential. Persistent lymphopenia strongly suggests a profound immunoparalysis or immunoexhaustion in T4 patients, which aligns with their baseline characteristics including older age, more comorbidities, and greater illness severity. “Immunosenescence” in older adults results in a global decline in lymphocyte counts and function, preventing effective immune reconstitution after sepsis and leading to persistent immunoparalysis [24–26]. Persistent immunoparalysis observed in T4 patients can be plausibly explained by the fact that their comorbidities (e.g., cardiovascular diseases, chronic kidney disease) have pre-exhausted the immune system’s responsiveness via upstream mechanisms such as chronic inflammation and immunosenescence [27]. Consequently, when sepsis strikes, their immune systems, lacking adequate functional reserve, are unable to effectively re-establish homeostasis. The utilization rate of vasoactive agents, sedatives, and analgesics in the T4 group was not the highest, instead, it was higher in the T2, which suggests that the poor long-term prognosis predicted by the T4 trajectory is not dominated by acute-phase hemodynamic collapse or insufficient treatment intensity.

It is worth noting that following the construction of multivariable Cox proportional hazards models, the difference in 7-day mortality between T4 and T1 was not significant, while the difference in 28-day mortality remained significant. In our previous study, we found both short-term and long-term mortality differed significantly in the general sepsis population. This might be explained by the fact that the selected patients in this study were already in a state of immunocompromise at baseline or had profound septic immune paralysis due to sepsis. Death within 7 days might be more driven by multiorgan dysfunction induced by the cytokine storm. The influence of “immune dynamic recovery potential,” reflected by the lymphocyte count trajectory, on short-term mortality might be masked by the “initial high-risk state,” which makes the short-term prognosis not differ significantly in T1 and T4 patients. The poor prognosis of the T4 population may be driven by multiple mechanisms. On one hand, there is a loss of key immunoregulatory and effector functions: the severe depletion of CD4 + T cells weakens their crucial “inflammatory brake” function, which is normally mediated by interactions with macrophage MHC-II molecules and negative regulation of pathways such as *TLR4/NF-κB* [28,29]. Meanwhile, surviving T cells enter an exhausted state, characterized by sustained high expression of inhibitory receptors such as PD-1, CTLA-4, TIM-3, and LAG-3 [8]. On the other hand, there is an expansion of immunosuppressive cell populations: MDSCs effectively suppress the activation of both CD4+ and CD8 + T cells and are significantly associated with an increased risk of nosocomial infections [30]. At the same time, the TNFR2 + Treg subset not only inhibits the proliferation of CD4 + T cells but also increases susceptibility to secondary pneumonia [31]. Furthermore, persistently elevated proportions of Tregs in non-surviving sepsis patients prevent immune recovery [32].

Furthermore, our trajectory plots demonstrate that by days 3–4 after sepsis onset, the T4 trajectory exhibited a distinct tendency to diverge from the other 3 trajectories, in which lymphocyte counts were close to normal and remained stable thereafter. The potential prognostic value of this specific time window is supported by clinical evidence linking early lymphocyte recovery to improved 28-day survival. For instance, a study focusing on elderly septic patients reported that failure to restore CD3 + T cell counts by days 3 and 7 was associated with significantly higher 28-day mortality, whereas the early recovery group had the lowest fatality rate [11]. Another study further confirmed that on day 4 post-sepsis, survivors had substantially higher lymphocyte counts than non-survivors, underscoring the critical significance of early immune reconstitution [33]. It is important to emphasize that recovery in lymphocyte quantity does not equate to functional restoration. Studies utilizing mouse models have demonstrated that although the numbers of CD4+ and CD8 + lymphocytes recover rapidly, the function of T helper (Th) cells against specific pathogens may be persistently impaired, resulting in the loss of pathogen-specific T cell clones [34].Notably, additional studies have explicitly verified that approximately 3–4 days after the onset of septic shock represents a pivotal transition period in the immune response, during which critically ill patients either regain homeostasis or progress to Persistent Inflammation, Immunosuppression, and Catabolism Syndrome (PICS) [35]. Consistently, a study investigating neutrophil functional alterations also designated “days 3–4” and “days 6–8” as core observation time points, which further highlights the clinical relevance of this time window for immune function assessment [36]. Collectively, these findings indicate that by days 3–4 after sepsis onset, patients’ immune status demonstrates stable trajectory characteristics with prognostic value. Consequently, this period represents the optimal window for initiating immunostimulatory therapy in the T4 subgroup.

### Limitations

This study has several limitations. First, although T4 (persistently low-level) served as a robust prognostic marker, it likely constituted a heterogeneous group. The absence of pre-ICU baseline data prevents us from distinguishing between acute lymphopenia induced by sepsis and pre-existing chronic lymphopenia. Second, the requirement for at least two lymphocyte count measurements within 7 days, essential for constructing the dynamic trajectory models, may introduce survival bias by excluding the most critically ill patients who died before a second lymphocyte measurement. Third, although the exclusion of patients with comorbidities such as malignancy or transplant status enhanced the internal validity of the study, it limits the generalizability of our findings to these frequently encountered patient populations. Finally, the lack of functional immunophenotyping data (e.g., lymphocyte subsets, cytokines) hinders a mechanistic exploration of the fundamental immune dysfunction underlying the identified trajectories, particularly T4. Our findings outline a clear research agenda to advance experimental and clinical studies for T4 patients, including prospective multicenter validation to enhance generalizability, elucidation of underlying mechanisms, and translation into precision clinical trials for immunoadjuvant therapies.

## Conclusion

In summary, this study represents a deepening of our research investigating immune dynamics in sepsis, shifting our perspective from “describing heterogeneity” to “precisely identifying the most vulnerable individuals within a high-risk subgroup.” By precisely targeting patients with lymphopenia, we first used GBTM to identify 4 distinct lymphocyte count trajectories, and identified T4 as a unique subgroup, which was confirmed as an independent risk factor for 28-day mortality. The T4 phenotype may be identifiable as early as days 3–4, defining a potential window for immunomodulatory intervention. Future research should build upon this enrichment strategy to prospectively validate the efficacy of targeted immunotherapies in the T4 population, ultimately advancing towards precision immunoadjuvant therapies, thereby potentially improving outcomes for sepsis patients.

## Supporting information

S1 Fig Kaplan-Meier curves depicting 7-day survival probabilities for the four distinct trajectory groups (T1-T4). (TIF)

S2 Fig Subgroup analysis of the association between lymphocyte trajectories and ICU mortality. (TIF)

S3 Fig Subgroup analysis of the association between lymphocyte trajectories and in-hospital mortality. (TIF)

S1 Table List of Abbreviations. (PDF)

S2 Table GBTM model selection criteria for lymphocyte trajectories. (PDF)

S3 Table 1640 Samples Missing Data. (XLSX)

S4 Table VIF Analysis Summary for Multiple Outcomes. (XLSX)
