# Supplementary material for: Failure of early lymphocyte recovery identifies sepsis patients with initial lymphopenia at highest risk for late mortality
Source: PLoS One. 2026 Jul 17;21(7):e0353698. doi: 10.1371/journal.pone.0353698 (PMC13378993; doi:10.1371/journal.pone.0353698)
Supplement: S1 Table — (PDF) [file pone.0353698.s004.pdf]

Abbreviation	Full Name
AIC	Akaike Information Criterion
AIDS	Acquired Immunodeficiency Syndrome
AKI	Acute Kidney Injury
ALP	Alkaline Phosphatase
ALT	Alanine Aminotransferase
ANOVA	Analysis of Variance
APACHE II	Acute Physiology and Chronic Health Evaluation II
APS III	Acute Physiology Score III
AST	Aspartate Aminotransferase
AvePP	Average Posterior Probability
BIC	Bayesian Information Criterion
BIDMC	Beth Israel Deaconess Medical Center
BUN	Blood Urea Nitrogen
CCI	Charlson Comorbidity Index
CD4+/CD8+	Cluster of Differentiation 4+/8+
CHF	Congestive Heart Failure
CI	Confidence Interval
CRRT	Continuous Renal Replacement Therapy
CTLA-4	Cytotoxic T-Lymphocyte-Associated Protein 4
DBP	Diastolic Blood Pressure
GBTM	Group-Based Trajectory Modeling
HR	Hazard Ratio
ICU	Intensive Care Unit
IL-6	Interleukin-6
INR	International Normalized Ratio
IQR	Interquartile Range
LAG-3	Lymphocyte-Activation Gene 3
LODS	Logistic Organ Dysfunction System
MAP	Mean Arterial Pressure
MDSCs	Myeloid-Derived Suppressor Cells
MHC-II	Major Histocompatibility Complex Class II
MIMIC-IV	Medical Information Mart for Intensive Care IV
MIT	Massachusetts Institute of Technology
NF-κB	Nuclear Factor Kappa-B
NLR	Neutrophil-to-Lymphocyte Ratio
OASIS	Oxford Acute Severity of Illness Score
OR	Odds Ratio
PCT	Procalcitonin
PD-1	Programmed Cell Death Protein 1
PICS	Persistent Inflammation, Immunosuppression, and Catabolism Syndrome
PT	Prothrombin Time
SAPS II	Simplified Acute Physiology Score II

SBP	Systolic Blood Pressure
SOFA	Sequential Organ Failure Assessment
STROBE	Strengthening the Reporting of Observational Studies in Epidemiology
Th	T helper (cell)
TIM-3	T-cell Immunoglobulin and Mucin-domain containing-3
TLR4	Toll-Like Receptor 4
TNFR2	Tumor Necrosis Factor Receptor 2
Tregs	Regulatory T cells
VIF	Variance Inflation Factor
WBC	White Blood Cell Count
