# Supplementary material for: Failure of early lymphocyte recovery identifies sepsis patients with initial lymphopenia at highest risk for late mortality
Source: PLoS One. 2026 Jul 17;21(7):e0353698. doi: 10.1371/journal.pone.0353698 (PMC13378993; doi:10.1371/journal.pone.0353698)
Supplement: S2 Table — (PDF) [file pone.0353698.s005.pdf]

S2 Table. GBTM model selection criteria for lymphocyte trajectories

trajectory	AIC	BIC	CAIC	SSBIC	HQIC	AvePP of each trajectory
1	8470	8507	8512	8491	8483	1
2	3114	3194	3206	3160	3141	0.97738/0.96337
3	1551	1677	1694	1623	1594	0.96452/0.96624/0.94059
4	833	1002	10253	929	890	0.96957/0.93799/0.95677/0.95493
5	892	1105	1134	1013	963	0.89757/0.95284/0.95555/0.91683/0.93266

AIC: Akaike Information Criterion; BIC: Bayesian Information criterion; CAIC: Consistent Akaike Information Criterion; SSBIC: Small-sample Bayesian Information Criterion; HQIC: Hannan-Quinn Information Criterion; SSBIC, Small-sample Bayesian Information Criter;Avepp: average posterior probability;
